# Gene Therapy for Hemoglobinopathies

**DOI:** 10.1089/hum.2018.122

**Published:** 2018-09-29

**Authors:** Marina Cavazzana, Fulvio Mavilio

**Affiliations:** ^1^ University of Paris Descartes-Sorbonne Paris Cité, IMAGINE Institute, Paris, France.; ^2^ Department of Life Sciences, University of Modena and Reggio Emilia, Modena, Italy.

**Keywords:** thalassemia, sickle-cell disease, globin genes, lentiviral vectors, gene editing

## Abstract

Gene therapy for β-thalassemia and sickle-cell disease is based on transplantation of genetically corrected, autologous hematopoietic stem cells. Preclinical and clinical studies have shown the safety and efficacy of this therapeutic approach, currently based on lentiviral vectors to transfer a β-globin gene under the transcriptional control of regulatory elements of the β-globin locus. Nevertheless, a number of factors are still limiting its efficacy, such as limited stem-cell dose and quality, suboptimal gene transfer efficiency and gene expression levels, and toxicity of myeloablative regimens. In addition, the cost and complexity of the current vector and cell manufacturing clearly limits its application to patients living in less favored countries, where hemoglobinopathies may reach endemic proportions. Gene-editing technology may provide a therapeutic alternative overcoming some of these limitations, though proving its safety and efficacy will most likely require extensive clinical investigation.

## Introduction

Hemoglobinopathies are inherited blood disorders characterized by defective synthesis of hemoglobin (Hb) chains or by the synthesis of mutated globin variants, such as the β^A-E6V^ causing sickle-cell diseases (SCD). Approximately 5% of the world population carries a Hb disorder trait, making hemoglobinopathies the most frequent monogenic diseases worldwide.^1^ The only therapy for β-thalassemia and SCD is allogeneic hematopoietic stem-cell (HSC) transplantation from human leukocyte antigen (HLA)-matched sibling donors.^2^ Matched unrelated or mismatched HSC transplants carry unacceptable risks of morbidity and mortality, given the current high standards of care. Transplantation of autologous, genetically corrected HSCs is a potential therapeutic alternative, carrying lower transplant-related risks and theoretically being available to all patients. Hematopoietic stem/progenitor cells (HSPCs) transduced by lentiviral vectors (LVs) have been used in clinical trials of gene therapy for immunodeficiencies and lysosomal storage disorders, providing strong evidence of safety and long-term efficacy in most cases.^3^ In particular. LVs showed relatively safe integration patterns in the human genome and appeared to cause little interference with normal gene regulation and no significant alterations in the homeostasis of the hematopoietic system.^4–6^

Gene therapy for hemoglobinopathies is more challenging. Regulation of globin gene expression is sophisticated and relies on the interaction of gene promoters with a locus-control region (LCR) that promotes high-level, erythroid-restricted transcription and controls the developmental switch of embryonic to fetal to adult globin gene expression.^7,8^ The combination of a full LCR and a complete β-globin gene is too large to fit in an LV: current vectors feature size-reduced LCR and non-coding portions of the β-globin gene but remain complex and transduce HSPCs less efficiently compared to simpler vectors, conflicting with the need to engraft a high proportion of transduced HSCs in the patient's bone marrow (BM). The clinical history of allogeneic HSC transplantation indicates that stable mixed chimerism with as low as 10–30% donor HSCs leads to significant amelioration of both β-thalassemia and SCD pathology, providing predictions about the minimal level of gene-corrected HSCs that may achieve clinical benefit.^9–11^ Recent gene therapy clinical trials are confirming these predictions, although the reduced output of a vector-borne transgene with respect to the natural β-globin locus significantly increases the minimal chimerism levels necessary to achieve efficacy, particularly in the case of SCD.^12–14^ Innovation in HSC procurement, transduction, and transplantation technology will likely increase the overall efficacy of gene therapy for hemoglobinopathies in the near future.

## LVs for Gene Therapy of Hemoglobinopathies

The first β-globin LVs were developed almost 20 years ago. They contained a β-globin gene under the control of the its promoter and 3′ enhancer elements in addition to the DNase I hypersensitive sites 2, 3, and 4 (HS2, HS3, and HS4) of the β-globin LCR.^15,16^ These vectors, termed TNS9 and HPV569, expressed levels of β-globin that were sufficient to correct murine models of β-thalassemia^15,17,18^ and SCD.^16^ The GLOBE vector had a different design, lacking the HS4 and the 3′ enhancer and featuring an extended HS2 and an HS3 element. This vector showed improved infectivity compared to vectors containing the HS4 and was equally efficacious in correcting murine β-thalassemia^19^ and reducing globin chain imbalance in erythroblasts from β-thalassemic patients.^20^ All globin vectors transduce long-term repopulating HSCs and combine the low genotoxic properties of LVs with the strict lineage specificity of the β-globin promoter, enhancer, and LCR.^21^

Attempts to increase globin gene expression by introducing larger HS fragments or adding the LCR HS1 element in the LV provided at best modest improvements.^22,23^ Other investigators added chromatin “insulators,” such as the chicken β-globin HS4^22,24^ or a synthetic element (FB) containing sequences from the chicken HS4 and the T-cell receptor BEAD-1 insulator, in an attempt to prevent repressive influence of surrounding chromatin on the integrated vector or reduce potential cis-acting influences of the LCR enhancers on neighboring genes.^25,26^ Unfortunately, these elements reduced transduction efficiency and caused genetic instability. In the first patient who received gene therapy for β-thalassemia, the chicken HS4 enhancer in the BGI vector contained a cryptic signal that triggered abnormal splicing of the proto-oncogene HMG2A and a benign clonal dominance.^12^ The element was removed for further clinical development.^27^ Other attempts to introduce chromatin remodeling elements, such as HS elements derived from the GATA-1 transcription factor gene^28^ or the ankyrin gene insulator,^29,30^ did improve vector expression features, indicating that combinations of erythroid-specific transcriptional regulatory elements may lead to more efficacious globin LVs. Nevertheless, the vectors currently in clinical development, such as BB305^21^ and GLOBE,^19^ contain just the essential promoter and LCR elements of the β-globin gene.

While a simple gene-replacement strategy may be sufficient to correct β-thalassemia, vectors aimed at correcting SCD must express globins able to compete with the β^S^-chain for α-chains and therefore interfere with Hb polymerization. The design of anti-sickling globins is based on genetic knowledge. SCD patients with elevated levels of fetal Hb (HbF), due to association with a hereditary persistence of HbF (HPFH) trait, experience less severe symptoms.^31,32^ The protective activity of HbF is due to the polymerization-inhibiting properties of γ-globin chains, which are able to inhibit sickling. Accordingly, LVs containing a γ-globin or a hybrid β-promoter/γ-globin gene efficiently corrected a murine model of SCD.^33,34^ Some investigators developed β-globins carrying mutations that interfere with axial and lateral contacts in the HbS polymer, with potent anti-sickling properties.^35^ The BB305 vector expresses one of these mutants, the β^A-T87Q^ globin, and is able to reduce sickling in a murine model of SCD.^16^ Another such mutant, the AS3 globin, carries three mutations—T87Q, G16D, and E22A—which further reduce polymerization while increasing the affinity for the α-chain.^36^ The βAS3-FB vector, which expresses AS3 globin, corrects murine SCD^37^ and reduces sickling in the erythroid progeny of CD34^+^ progenitors from SCD patients.^26,38^ The BB305 and βAS3-FB vectors are currently in clinical development for gene therapy of SCD.

## Gene Therapy for β-Thalassemia

Beta thalassemia is caused by mutations that reduce (β^+^) or abolish (β^0^) the synthesis of β-globin chains,^39^ resulting in ineffective erythropoiesis, intramedullary hemolysis, and hemolytic anemia. Clinical severity is variable and depends on the combination of different β^0^ or β^+^ mutations. The association with α-thalassemia or elevated HbF levels cause milder clinical phenotypes.^40,41^ Patients with severe β-thalassemia present with anemia, iron overload, hepatosplenomegaly, and skeletal abnormalities due to the BM expansion. Complications include cardiopathy, hepatic dysfunction, and endocrine disorders, which occur at various ages, depending on disease severity and adequacy of the transfusion and iron chelation treatment.^42^ At present, the only cure for β-thalassemia is allogeneic HSC transplantation from HLA-identical donors, with implies high-dose chemotherapy and immunosuppression.^43^ Transplantation with HSCs from a matched sibling donor results in >80% disease-free survival and 3–10% mortality on long-term follow-up,^2,44,45^ but is limited by a donor availability of <30%.^46^ Transplants from matched unrelated donors result in reduced disease-free survival and increased mortality,^47^ while haploidentical transplantation carries unacceptably high risks. Chronic graft-versus-host disease, infections, and the toxicity associated with BM conditioning are common side effects of allogeneic HSC transplantation and affect both quality of life and survival. Gene therapy is theoretically available to all patients and, due to the autologous origin of the transplanted cells, is associated with reduced toxicity and treatment-related morbidity.

The traditional sources of stem cells for gene therapy are BM or peripheral blood mobilized CD34^+^ HSPCs. Mobilization has essentially replaced BM harvest due to higher yield of CD34^+^ cells, faster engraftment, enhanced immune reconstitution, and shorter hospitalization. The issue of HSC procurement is critical, since the minimal target dose of CD34^+^ (usually >5 × 10^6^ cells/kg) is difficult to obtain by BM harvesting. Mobilization in the peripheral blood by administration of granulocyte-colony stimulating factor (G-CSF) provides adequate doses of CD34^+^ HSPCs in most conditions but may be risky in β-thalassemia patients.^48^ An alternative agent is Plerixafor (AMD3100; Mozobil™), a bicyclam molecule that antagonizes the binding of stromal cell-derived factor 1 to the chemokine CXC-receptor-4, causing mobilization of HSPCs in the peripheral circulation.^49^ The use of Plerixafor in combination with G-CSF is currently approved in the United States and Europe for adult and pediatric populations.^50^ Plerixafor, or G-CSF + Plerixafor, safely mobilizes adequate numbers of HSCs in β-thalassemia patients.^51–53^ Plerixafor alone mobilizes HSCs with superior stem-cell characteristics, although with a somewhat lower yield compared to the association with G-CSF.^54^ In the currently used protocols, mobilized CD34^+^ cell populations are transduced by LVs and readministered to patients by intravenous or intrabone infusion. Adequate cell doses and clonal diversity of engrafted cells are necessary to reconstitute hematopoiesis rapidly and completely after transplantation, requiring high transduction efficiency and fully myeloablative conditioning regimens. The morbidity associated with conditioning is reduced in gene therapy compared to allogeneic HSC transplantation, since immune suppression is not required in an autologous setting.

The potential efficacy of HSC transduced with β-globin LVs in correcting the β-thalassemia phenotype was proven in murine models of the disease, starting from a pioneering study in 2000.^15,18,19,55–57^ Interestingly, one of these studies showed an *in vivo* survival advantage for genetically corrected erythroblasts in a murine β-thalassemia model,^19^ predicting that even suboptimal doses or transduced HSC may provide clinical benefit in patients. The potential genotoxicity of the currently used BB305 and GLOBE vectors was tested more recently in rigorous studies that fully supported clinical application.^21,58^ Clinical development of gene therapy started in 2007 with the transplantation of HSCs transduced by the BGI LV expressing the β^T87Q^ globin in a patient affected by transfusion-dependent HbE/β-thalassemia.^12^ The HbE trait mimics a mild β^+^ allele and reduces the requirement for therapeutic β-globin synthesis. The patient received myeloablative busulfan conditioning, experienced a gradual increase in gene-marked cells up to 10–20%, and became transfusion independent with stable Hb levels of 8.5–9 g/dL 1 year after gene therapy. The mutant HbA, HbF, and HbE contributed almost equally to the therapeutic Hb levels.^12^ Interestingly, the synthesis of therapeutic globin was largely accounted for by the expansion of a single stem/progenitor cell clone in which the LV integrated into the *HMGA2* proto-oncogene. The benign dominant clone persisted for almost 9 years, before declining to <10% of the circulating nucleated cells. The patient currently maintains stable levels of therapeutic Hb and requires only occasional transfusion (M. Cavazzana, unpublished observations). This pilot study proved the efficacy of gene therapy for β-thalassemia, but also indicated the requirement of an abundant, polyclonal population of transduced HSCs for significant and long-lasting clinical benefit. A clinical trial carried out in the United States using partially myeloablative conditioning showed minimal clinical benefit, confirming the requirement for full myeloablation for efficient engraftment of genetically corrected HSCs.^59^

Two subsequent clinical trials addressing transfusion-dependent β-thalassemia started in 2013 in the United States, Australia, Thailand, and France based on the use of the BB305 vector. At a median follow-up of 26 months, all but one of the 13 patients with a non β^0^/β^0^ thalassemia genotype had discontinued red cell transfusions and remained transfusion-independent with levels of total Hb of 8.2–13.7 g/dL, of which the therapeutic HbA^T87Q^ accounted for 3.4–10 g/dL. In nine patients with β^0^/β^0^ or severe β^+^/β^+^ (homozygous IVS1-110 mutation) genotypes, the median annualized transfusion volume decreased by 73%, and transfusions were discontinued in three patients. The treatment was well tolerated, with no severe adverse event related to gene transfer. Vector integration analysis showed polyclonal hematopoietic reconstitution and no evidence of vector-related clonal dominance.^14^ These studies proved the safety and remarkable efficiency of LV-mediated gene therapy in reducing or eliminating the transfusion dependence in β-thalassemia patients.

Mobilization of HSPCs with Plerixafor + G-CSF was introduced in a Phase I/II clinical trial carried out in Italy addressing transfusion-dependent β-thalassemia. The study included three cohorts of adult (≥18 years; *n* = 3), adolescent (8–17 years; *n* = 3), and pediatric (3–7 years; *n* = 4) subjects. Mobilized CD34^+^ cells were transduced with the GLOBE vector and administered by intra-osseous injection in the posterior-superior iliac crests after myeloablative conditioning with treosulfan and thiotepa, with the rationale of favoring homing and engraftment.^60^ As of March 2018, nine patients with different genotypes (β^0^/β^0^, β^+^/β^+^, and β^0^/β^+^) were treated, and the procedure was well tolerated, with no treatment-related adverse events. All tested patients showed multilineage cell engraftment and no evidence of clonal abnormality. Transfusion requirement was significantly reduced in the three adult patients, while three out of four evaluable pediatric participants discontinued transfusions and remained transfusion independent, with a follow-up of >12 months.^61^

Overall, the clinical trials (see list in Table 1) showed that clinical efficacy is directly correlated with gene transfer efficiency and the dose of genetically corrected HSCs and inversely correlated with the Hb synthesis requirement, where β^0^ mutations are more difficult to correct than β^+^ ones. Clinical efficacy also correlated with the average proviral copy number in transduced cells, and therefore with the overall output of therapeutic β-globin. In all trials, cell manufacturing appeared to be a critical factor, where continuous improvements in transduction conditions will play a major role in increasing efficacy and make gene therapy the treatment of choice for β-thalassemia patients.

**Table 1.** Active gene therapy clinical trials for β-thalassemia

*Country*	*Sponsor*	*Gene*	*Vector*	*Conditioning*	*Enrolment*	*Identifier*	*Status*
United States	MSKCC	β-globin	TNS9.3.55	Busulfan: 8 mg/kg	Adults, 10 patients	NCT01639690	Active: 4 patients treated; not recruiting
France	Bluebird Bio	β^A-T87Q^ globin	BB305	Busulfan: 12.8 mg/kg, pk-adjusted	Age 5–37 years, 7 patients	NCT02151526	Active: 4 patients treated; not recruiting
United States, Thailand, Australia	Bluebird Bio	β^A-T87Q^ globin	BB305	Busulfan: 12.8 mg/kg	Adults ≤50 years, 23 patients	NCT02906202	Active: 18 patients treated; recruiting
Italy	Telethon Foundation	β-globin	GLOBE	Treosulfan 42 g/m^2^ + Thiotepa 8 mg/kg	Age 3–64 years, 10 patients	NCT02453477	Active: 9 patients treated; recruiting

## Gene Therapy for Scd

SCD is caused by a mutation (E6V) in the β-globin chain that induces polymerization of Hb tetramers upon deoxygenation. Hb polymers cause erythrocytes to adopt the characteristic sickle shape that reduces their flexibility in the capillary circulation, causing ischemia, multi-organ damage, severe pain, hemolytic anemia, and stroke, significantly shortening the patients' life-span.^7,62^ Current treatments include periodical transfusion and administration of hydroxyurea, which moderately increases HbF synthesis.^62,63^ Also for SCD, the only definitive treatment is allogeneic HSC transplantation from matched related donors, with a reported >90% disease-free survival over 6 years.^2^ However, HSC transplantation is not frequently performed in SCD patients, given the toxicity and potential sterility associated with conditioning and the difficulty in finding suitable donors. As for β-thalassemia, gene therapy is a less toxic therapeutic option potentially available to all patients, though cell procurement and engraftment is even more challenging in the case of SCD.

Preclinical studies showed that LV-based gene therapy is potentially efficacious in reducing sickling. The vectors used in these studies contained the same combination of β-globin promoter and LCR elements used for β-thalassemia, and express anti-sickling globins such as the fetal γ-globin,^33,34^ the β^T87Q^,^16^ or the βAS3^26,37,38^ mutants. As for β-thalassemia, allogeneic HSC transplantation showed that stable mixed chimerism with donor HSC levels of 20–30% is sufficient to achieve significant hematologic and clinical improvement.^9–11^ Combined with the evidence that the association of SCD and HPFH results in a milder phenotype, these data predict that engraftment of >20% of HSC producing RBCs with >30% anti-sickling Hb levels could improve symptoms and reduce transfusion requirement.

Clinical development of gene therapy for SCD started with a first patient treated in France, with transplantation of CD34^+^ HSPCs transduced with the BB305 vector expressing the β^A-T87Q^ globin at a dose of 5 × 10^6^ cells/kg and an average vector copy number (VCN) of 1 after full myeloablative conditioning. The patient rapidly reconstituted a polyclonal hematopoiesis, achieved transfusion independence with a level of β^A-T87Q^ globin of ∼50%, and experienced no treatment-related adverse event over a 2-year follow-up.^13^ These results prompted the initiation of two clinical trials in France and the United States on severe SCD patients. However, the multicenter U.S. trial failed to reproduce the success of the first patient. The first seven patients treated received a median dose of 2 × 10^6^ CD34^+^ BM-derived HSPCs, with a median VCN of 0.6. A follow-up of 8–17 months showed a VCN in the peripheral blood of <0.12, HbA^T87Q^ levels of <2 g/dL, and an average HbA^T87Q^/HbS ratio of <15%.^64^ These results showed that mobilization and robust engraftment of transduced HSCs is more difficult to achieve in SCD than in β-thalassemia patients. Cell dose, transduction efficiency, and engraftment are apparently limiting factors in achieving the minimal HSC chimerism and synthesis of anti-sickling Hb in erythroblasts predicted to provide clinical benefit.

Harvesting CD34^+^ cells is a risky procedure in SCD patients, which often fails to provide an adequate cell dose. In this regard, the BM microenvironment appears to be a critical factor. Capillary occlusion caused by sickling leads to ischemia, inflammation, and oxidative stress,^65^ which compromise the quality of the HSC niche and impact on both harvest and re-engraftments of HSCs. Attempts to mobilize HSCs by low doses of G-CSF caused severe adverse events and at least one fatality, and was therefore abandoned.^66^ A Plerixafor-only mobilization trial recently completed in France proved the safety of the drug in an SCD context and provided an estimate of the yield of CD34^+^ HSPCs in both adult and juvenile patients.^67^ Plerixafor mobilization has been recently implemented in the ongoing gene therapy clinical trials (see list in Table 2). A better cell quality and dose, together with ongoing innovation in cell manufacturing to increase transduction efficiency, should rapidly improve the clinical outcome of gene therapy for SCD.

**Table 2.** Active gene therapy clinical trials for sickle-cell disease

*Country*	*Sponsor*	*Gene*	*Vector*	*Conditioning (TD)*	*Enrolment*	*Identifier*	*Status*
France	Bluebird Bio	β^A-T87Q^ globin	BB305	Busulfan: 12.8 mg/kg, pk-adjusted	Age 5–37 years, 7 patients	NCT02151526	Active: 3 patients treated; not recruiting
United States	Bluebird Bio	β^A-T87Q^ globin	BB305	Busulfan: 12.8 mg/kg	Adults, 29 patients	NCT02140554	Active: 9 patients treated
United States	UCLA	βAS3 globin	βAS3-FB	Busulfan: 12.8 mg/kg, pk-adjusted	Adults, 6 patients	NCT02247843	Active: 1 patient treated; recruiting
United States, Jamaica	Cincinnati Children's Hospital	γ-globin	mLARβΔγV5	Melphalan: 140 mg/m^2^	Age 18–35 years, 10 patients	NCT02186418	Active: recruiting
United States	Boston Children's Hospital	BCL11A shRNA^mir^	LCR-shRNA^mir^	Busulfan: 12.8 mg/kg, pk-adjusted	Age 3–40 years, 7 patients	NCT03282656	Active, 1 patient treated; recruiting

## Gene Therapy for Hemoglobinopathies: Future Directions

A limitation of the *ex vivo* gene therapy approach for hemoglobinopathies is the complex, difficult, and very expensive cell manufacturing step. Administering a globin vector directly *in vivo* would make gene therapy easier to perform and more feasible in less favored country, where cost and complexity represent formidable barrier to patient access. Approaches based on the use of capsid-modified adenovirus vectors and the integration-promoting machinery of the *Sleeping Beauty* transposon have been recently published^68^ and may represent a promising alternative to LVs to integrate a β-globin expression cassette in repopulating stem cells directly into the BM.

Gene editing has recently emerged as a potential alternative to vector-mediated gene addition for gene therapy of β-hemoglobinopathies. Editing the β^S^ mutation by homology-directed DNA repair (HDR) may be achieved by using zinc-finger nucleases or CRISPR/Cas9 to cleave the β^S^ locus and viral genomes or single-stranded oligonucleotides as HDR donor templates. Correction of the β^S^ mutation can be obtained in CD34^+^ HSPCs with remarkable efficiency, achieving potentially therapeutic HbA/HbS ratios in the erythroid progeny of edited cells differentiated *in vitro* or *in vivo* in NOD SCID gamma mice.^69–72^

An alternative strategy aims at reactivating HbF synthesis in adult erythroblasts. A first approach is based on downregulating BCL11A, a transcription factor involved in silencing γ-globin expression in adult erythroblasts.^73,74^ Downregulating BCL11A by restricted expression of an shRNA in the erythroid compartment after delivery to HSCs by a GLOBE-like vector is a potentially efficacious strategy^75^ that recently entered clinical investigation (see Table 2). Fine modulation of the shRNA is, however, necessary, since BCL11A is essential for HSC and lymphoid cell development^76^ and may be required for RBC enucleation.^77^ Alternatively, BCL11A can be down-modulated by deleting the erythroid-specific enhancers controlling its erythrocyte-restricted expression by CRISPR/Cas9-mediated genome editing.^78,79^ A second approach is based on recreating genomic deletions or mutations in the β-globin locus associated with HPFH,^7,32^ leading to increased γ-globin expression^80–82^ although with sub-therapeutic efficiency compared to LV-mediated gene replacement. This strategy is less complex than editing the β^S^ mutation, since it relies on non-homologous end joining, the dominant DNA repair pathway in HSPCs,^69,83^ and does not require delivering a DNA template. A more creative approach is based on the use of ZF protein coupled to an effector domain, inducing a loop between the LCR and the γ-globin promoters, which reactivates γ-globin synthesis at the expenses of β^S^-globin without requiring DNA cleavage.^84^

Editing the genome holds great promises for gene therapy of hemoglobinopathies and may offer advantages with respect to LV-mediated gene replacement in terms of complexity and cost of manufacturing genetically modified HSCs. However, this technology has still to prove its efficacy and biosafety profile in a real-life context. Off-target effects and unrepaired double-stranded cuts may represent significant risk factors, not necessarily inferior to the potential genotoxic effects of LV integration in the human genome. Given the absence of reliable preclinical models to analyze editing efficiency in long-term repopulating HSCs and any associated genotoxicity, the efficacy and safety of gene editing may eventually be proven only in clinical trials.
